# Association of Serum Cholesterol Levels With Peripheral Nerve Damage in Patients With Type 2 Diabetes

**DOI:** 10.1001/jamanetworkopen.2019.4798

**Published:** 2019-05-31

**Authors:** Johann M. E. Jende, Jan B. Groener, Christian Rother, Zoltan Kender, Artur Hahn, Tim Hilgenfeld, Alexander Juerchott, Fabian Preisner, Sabine Heiland, Stefan Kopf, Mirko Pham, Peter Nawroth, Martin Bendszus, Felix T. Kurz

**Affiliations:** 1 Department of Neuroradiology, Heidelberg University Hospital, Heidelberg, Germany; 2 Department of Endocrinology, Diabetology and Clinical Chemistry (Internal Medicine 1), Heidelberg University Hospital, Heidelberg, Germany; 3 German Center of Diabetes Research (DZD), München-Neuherberg, Germany; 4 Division of Experimental Radiology, Department of Neuroradiology, Heidelberg University Hospital, Heidelberg, Germany; 5 Department of Neuroradiology, Würzburg University Hospital, Würzburg, Germany; 6 Institute for Diabetes and Cancer, Helmholtz Diabetes Center, Helmholtz Center Munich, Munich, Germany

## Abstract

**Importance:**

Lowering serum cholesterol levels is a well-established treatment for dyslipidemia in patients with type 2 diabetes (T2D). However, nerve lesions in patients with T2D increase with lower serum cholesterol levels, suggesting that lowering serum cholesterol levels is associated with diabetic polyneuropathy (DPN) in patients with T2D.

**Objective:**

To investigate whether there is an association between serum cholesterol levels and peripheral nerve lesions in patients with T2D with and without DPN.

**Design, Setting, and Participants:**

This single-center, cross-sectional, prospective cohort study was performed from June 1, 2015, to March 31, 2018. Observers were blinded to clinical data. A total of 256 participants were approached, of whom 156 were excluded. A total of 100 participants consented to undergo magnetic resonance neurography of the right leg at the Department of Neuroradiology and clinical, serologic, and electrophysiologic assessment at the Department of Endocrinology, Heidelberg University Hospital, Heidelberg, Germany.

**Exposures:**

Quantification of the nerve’s diameter and lipid equivalent lesion (LEL) load with a subsequent analysis of all acquired clinical and serologic data with use of 3.0-T magnetic resonance neurography of the right leg with 3-dimensional reconstruction of the sciatic nerve.

**Main Outcomes and Measures:**

The primary outcome was lesion load and extension. Secondary outcomes were clinical, serologic, and electrophysiologic findings.

**Results:**

A total of 100 participants with T2D (mean [SD] age, 64.6 [0.9] years; 68 [68.0%] male) participated in the study. The LEL load correlated positively with the nerve’s mean cross-sectional area (*r* = 0.44; *P* < .001) and the maximum length of a lesion (*r* = 0.71; *P* < .001). The LEL load was negatively associated with total serum cholesterol level (*r* = −0.41; *P* < .001), high-density lipoprotein cholesterol level (*r* = −0.30; *P* = .006), low-density lipoprotein cholesterol level (*r* = −0.33; *P* = .003), nerve conduction velocities of the tibial (*r* = −0.33; *P* = .01) and peroneal (*r* = −0.51; *P* < .001) nerves, and nerve conduction amplitudes of the tibial (*r* = −0.31; *P* = .02) and peroneal (*r* = −0.28; *P* = .03) nerves.

**Conclusions and Relevance:**

The findings suggest that lowering serum cholesterol levels in patients with T2D and DPN is associated with a higher amount of nerve lesions and declining nerve conduction velocities and amplitudes. These findings may be relevant to emerging therapies that promote an aggressive lowering of serum cholesterol levels in patients with T2D.

## Key Points

**Question:**

Is there an association between a low serum cholesterol level and the extent of peripheral nerve damage as assessed with magnetic resonance neurography in patients with type 2 diabetes?

**Findings:**

In this cross-sectional cohort study of 100 adults with type 2 diabetes, the amount of nerve lesions was negatively associated with total serum cholesterol levels.

**Meaning:**

The findings suggest that lowering serum cholesterol levels in patients with type 2 diabetes is associated with diabetic polyneuropathy.

## Teaser

This study investigates whether an association exists between serum cholesterol levels and peripheral nerve lesions in patients with type 2 diabetes with and without diabetic polyneuropathy.

## Introduction

Distal symmetric diabetic polyneuropathy (DPN) is one of the most severe complications of diabetes, affecting approximately 200 million patients worldwide, with increasing prevalence leading to high morbidity and rising health care costs.^1^ Although the exact metabolic processes underlying DPN are still uncertain, several clinical and serologic risk factors for developing DPN, such as obesity, hypertension, hyperglycemia, dyslipidemia, and a decrease in renal function, have been identified in clinical studies.^2,3,4^ The poor outcomes after adjusting serum glucose levels in patients with type 2 diabetes (T2D) compared with type 1 diabetes (T1D) suggest that risk factors other than hyperglycemia might play an important role in the development of DPN.^3,5^ An in vitro study^6^ found that, in samples from patients with T2D and DPN, lipid composition of Schwann cells is altered compared with samples from control individuals without DPN. To date, it has not been possible to visualize those alterations in the nerve’s microstructure in vivo. Inpatient magnetic resonance neurography (MRN) at 3.0 T is a noninvasive method that allows for an exact qualitative and quantitative analysis of nerve damage in different neuropathies.^7,8,9^ Recent results from an MRN study^10^ in patients with DPN have shown that a decrease in serum high-density lipoprotein cholesterol (HDL-C) levels is associated with an increase in fat-equivalent lesions of the sciatic nerve and an increase in clinical symptom severity. These nerve lesions also occurred more frequently in patients with T2D compared with patients with T1D.^10^ The exact role of cholesterol metabolism in the development of DPN, however, is unknown, and it has not yet been determined whether lowering serum cholesterol levels in patients with T2D has a positive influence on the course of T2D DPN.^11^ Some clinical studies^11,12^ have found that a lowering of serum cholesterol levels had positive effects on the course of DPN that were mainly attributed to lowering low-density lipoprotein cholesterol (LDL-C) levels and to anti-inflammatory and antioxidative effects of statin treatment. However, low serum cholesterol levels are associated with neuropathic symptoms and impair nerve regeneration after axonal damage in neurons of the central and peripheral nervous systems.^13,14,15,16,17^ This association was mainly attributed to an insufficient supply of cholesterol to neurite tips and adjacent Schwann cells of regenerating axons as a consequence of a decrease in lipoproteins.^14,18,19,20,21^ With regard to emerging therapies, such as protein convertase subtilisin/kexin type 9 (PCSK9) inhibitors that promote an aggressive lowering of total serum cholesterol levels,^16^ it is crucial to understand whether a decrease in total serum cholesterol and LDL-C levels is beneficial or potentially harmful for patients with T2D with DPN.

The aim of this study was to investigate the association of cholesterol metabolism in combination with other potential clinical and serologic risk factors with the development of macrostructural and microstructural alterations of the sciatic nerve in T2D. To acquire factors such as the mean cross-sectional area (MCA) of the sciatic nerve and to achieve a precise calculation of volume and extent of nerve lesions in relation to vital nerve tissue, this study used high-resolution MRN at 3.0 T in combination with advanced image analysis tools.

## Methods

### Study Design and Participants

Participants were screened and recruited at the outpatient clinic of the Department of Endocrinology, Heidelberg University Hospital, Heidelberg, Germany, where all clinical, serologic, and electrophysiologic examinations were performed. Thereafter, patients underwent MRN at the Department of Neuroradiology, where image processing was performed. All patient data were pseudonymized, and participating researchers at the Department of Neuroradiology were blinded to all patient data. In total, 256 patients were screened, and 156 were excluded. A total of 100 patients with T2D took part in this prospective study from June 1, 2015, to March 31, 2018. Overall exclusion criteria were age younger than 18 years; pregnancy; any contraindications for magnetic resonance imaging; any history of lumbar surgery; relevant disc protrusion or herniation; any other risk factors for neuropathy, such as alcoholism, malignant or infectious diseases, hypovitaminosis, or monoclonal gammopathy; any previous or ongoing exposure to neurotoxic agents; and any chronic neurologic diseases, such as Parkinson disease, restless leg syndrome, or multiple sclerosis. To exclude severe renal insufficiency or macroangiopathy as potential confounders, only patients with an estimated glomerular filtration rate (eGFR) greater than 60 mL/min, an ankle-brachial index (ABI) greater than 0.9, and an intima-media thickness (IMT) less than 0.9 mm were included in the study. This study was approved by the Heidelberg Study on Diabetes and Complications Ethics Committee, and all participants gave written informed consent. The study followed the Strengthening the Reporting of Observational Studies in Epidemiology (STROBE) reporting guideline.

### Clinical and Electrophysiologic Examination

For every patient, a detailed medical history was documented and an examination of neuropathic symptoms was performed according to the guidelines issued by the German Society for Diabetology, including evaluation of the neuropathy deficit score (NDS) and the neuropathy symptom score (NSS).^22^

DPN was determined according to the following criteria: a score of 5 or higher in the NDS or NSS (if a discrepancy between the NDS and NSS was found, the higher score was chosen^23^) and abnormal nerve conduction test results in at least 2 different nerves.

The electrophysiologic examination (Viasys Healthcare VikingQuest; Viasys Healthcare GmbH) of the right leg included the following: distal motor latencies of the right tibial and peroneal nerves; motor (compound muscle action potentials [CMAPs]) and sensory amplitudes (sensory nerve action potential) of the tibial, peroneal, and sural nerves; and nerve conduction velocities (NCVs) of the tibial, peroneal, and sural nerves. The skin temperature was at least 32 °C throughout the examination. The 24-hour blood pressure was documented (TM-2430 with CA11 blood pressure cuff, size adapted to the patient’s upper arm circumference; Boso d.o.o.), and IMT was detected with duplex ultrasonographic examination of both carotid arteries (SonoAce X8; Samsung Group). The ABI was calculated using noninvasive blood pressure measurements of the arms and ankles (ABI System 1000; Boso d.o.o.).

Blood samples were obtained while patients were in a fasting state and processed immediately under standardized conditions in the central laboratory of Heidelberg University Hospital. Albumin excretion in urine was detected in morning spot urine within all participants. Estimated glomerular filtration rate was calculated with the Chronic Kidney Disease Epidemiology Collaboration formula.^24^

### MRN Imaging Protocol

**Figure 1.**
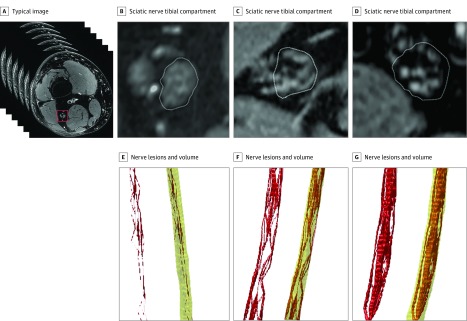
Segmentation of Sciatic Nerve Hypointense Lesions in a T2-Weighed, Fat-Suppressed Sequence and Subsequent Processing of Image Factors A, Typical acquired image at the thigh level. Red box indicates the sciatic nerve. B-D, Examples of different amounts of nerve lesions and clinical factors for 3 different patients at different clinical diabetic polyneuropathy stages. B, No polyneuropathy (neuropathy symptom score [NSS] = 0, neuropathy deficit score [NDS] = 5, total serum cholesterol [SC] level = 220 mg/dL [to convert to millimoles per liter, multiply by 0.0259). C, Moderate polyneuropathy (NSS = 4, NDS = 6, total SC = 167 mg/dL). D, Severe polyneuropathy (NSS = 6, NDS = 7, total SC = 40 mg/dL). E-G, Three-dimensional reconstructions of nerve lesions (red) and vital nerve volume (yellow) for the 3 patients with type 2 diabetes in panels B through D. E, No polyneuropathy (patient in B): few nerve lesions. F, Moderate polyneuropathy (patient in C): moderate amount of nerve lesions. G, Severe polyneuropathy (patient in D): extensive nerve lesions. Additional details about statistical correlations of lesion load or length are in the Results section.

All participants underwent high-resolution MRN of the right leg in a 3.0-T magnetic resonance scanner (Magnetom TIM-TRIO; Siemens Healthcare). A 15-channel transmit-receive extremity coil was used, and we applied an axial, high-resolution, T2-weighted, turbo spin-echo, 2-dimensional sequence with spectral fat saturation (T2wFS) of the right middle thigh at the following settings: relaxation time, 5970 milliseconds; echo time, 55 milliseconds; field of view, 160 × 160 mm^2^; matrix size, 512 × 512; section thickness, 4 mm; intersection gap, 0.35 mm; voxel size, 0.5 × 0.3 × 4.0 mm^3^; and number of sections, 24. No artificial image filters were used to avoid artificial alteration of the acquired T2wFS signal (Figure 1A gives a typical acquired image at the thigh level). In each participant, the sequence was centered to the sciatic nerve bifurcation to ascertain that the anatomical region mapped by MRN was comparable in all participants.

### Image Postprocessing 

All images generated were pseudonymized. Images at the middle thigh level were analyzed in a semiautomatic approach using ImageJ and custom-written code in Matlab, version 7.14.0.0739 (R2012a).^25,26^ A total number of 2400 images were analyzed accordingly. Anatomical segmentation of the sciatic nerve’s tibial compartment and the proximal tibial nerve was performed for all participants. The peroneal compartment was excluded from nerve segmentation because its curving course at the distal thigh level below the sciatic nerve’s bifurcation poses a potential risk for magic angle artifacts and does not allow for precise volumetric analysis based on axial T2wFS images.^27,28^ Lesions were segmented by comparing muscle and nerve signals on every individual section. The detailed process of anatomical nerve segmentation and lesion mapping has been described elsewhere.^10^ Binarized maps of lesions and vital nerve tissue were analyzed in Matlab^26^ (Figure 1B-D gives a 3-dimensional reconstruction of T2wFS-hypointense tibial nerve lesions in 3 patients with T2D at different clinical stages of DPN). Specifically, we determined the lesion ratio as the number of lesion voxels divided by the number of voxels in vital nerve tissue. We further obtained the MCA and the maximal craniocaudal length of a lesion of each nerve using lesion-specific, 3-dimensional, connected-component labeling with a voxel connectivity of 26.^28^

### Statistical Analysis

Statistical data analysis was performed with GraphPad Prism 6 (GraphPad Software Inc). All data were tested for gaussian normal distribution using the D’Agostino-Pearson omnibus normality test. If a gaussian normal distribution was given, *t* tests were used for comparisons of 2 groups, 1-way analyses of variance were applied for comparisons of more than 2 groups, and Bonferroni-corrected Pearson correlation coefficients were calculated for correlation analysis. If data were not gaussian distributed, the Mann-Whitney test was used for comparisons of 2 groups, the Kruskal-Wallis test was used for multiple comparisons of more than 3 groups, and Dunn-corrected nonparametric Spearman correlations were applied for correlation analysis. For all tests, the level of significance was defined at a 2-tailed *P* < .05. All results are presented as mean (SE).

## Results

### Clinical and Epidemiologic Data

A total of 100 patients with T2D with DPN (n = 64) or without DPN (n = 36) were included in this study (mean [SD] age, 64.6 [0.9] years; 68 [68.0%] male). Total serum cholesterol level was positively correlated with tibial NCV (*r* = 0.32; *P* = .02), peroneal NCV (*r* = 0.30; *P* = .03), and tibial nerve CMAP (*r* = 0.35; *P* = .01). Serum LDL-C level was also positively correlated with tibial NCV (*r* = 0.28; *P* = .04), peroneal NCV (*r* = 0.30; *P* = .02), and tibial CMAP (*r* = 0.44; *P* = .001). No such correlation was found for serum HDL-C level. Furthermore, no correlations were found for patient’s age, body mass index (BMI), blood pressure, glycosylated hemoglobin (HbA_1c_) levels, or renal function outcomes with electrophysiologic findings or clinical scores. Clinical, electrophysiologic, and serologic data of patients with and without DPN are presented in Table 1.

**Table 1.** Comparison of Magnetic Resonance Neurography Findings With Demographic, Serologic, and Electrophysiologic Data in Patients With and Without Diabetic Neuropathy

Variable	Mean (SE)		*P* Value
	Diabetic Neuropathy	No Diabetic Neuropathy	
Lesion load in vital nerve tissue, %	19.67 (2.03)	10.03 (0.87)	<.001
Maximum length of a lesion, mm	63.47 (2.44)	50.07 (3.26)	.001
Mean cross-sectional area of the tibial nerve, mm^3^	148.20 (5.24)	122.20 (3.82)	<.001
Age, y	67.49 (1.09)	61.43 (1.56)	.006
Disease duration, y	12.81 (1.35)	9.22 (1.20)	.05
BMI	29.59 (0.79)	30.35 (0.81)	.50
NSS	5.18 (0.43)	2.68 (0.48)	<.001
NDS	5.16 (0.43)	2.33 (0.35)	<.001
Total serum cholesterol level, mg/dL	175.00 (6.32)	197.31 (6.27)	.02
LDL-C level, mg/dL	87.73 (4.57)	113.90 (5.51)	<.001
HDL-C level, mg/dL	52.21 (3.52)	51.14 (2.35)	.81
Triglyceride levels, mg/dL	221.92 (29.78)	166.71 (11.94)	.11
HbA_1c_, %	7.40 (0.54)	6.91 (0.18)	.42
Creatinine level, mg/dL	0.92 (0.04)	0.83 (0.05)	.13
eGFR, mL/min	80.46 (3.15)	89.72 (2.96)	.07
Sural			
NCV, m/s	43.19 (4.22)	44.18 (1.79)	.81
SNAP, mV	4.98 (0.94)	5.67 (0.56)	.51
Peroneal			
NCV, m/s	36.62 (1.08)	42.64 (0.98)	<.001
SNAP, mV	3.16 (0.42)	5.90 (0.46)	<.001
DML, ms	5.79 (0.55)	4.11 (0.10)	.007
Tibial			
NCV, m/s	37.72 (1.36)	42.35 (0.82)	.007
CMAP, mV	6.62 (0.95)	13.53 (1.01)	<.001
DML, ms	6.27 (0.72)	5.26 (0.66)	.31

Abbreviations: BMI, body mass index (calculated as weight in kilograms divided by height in meters squared); CMAP, compound muscle action potential; DML, distal motor latency; eGFR, estimated glomerular filtration rate; HbA_1c_, hemoglobin A_1c_; HDL-C, high-density lipoprotein cholesterol; LDL-C, low-density lipoprotein cholesterol; NCV, nerve conduction velocity; NDS, neuropathy disability score; NSS, neuropathy symptom score; SNAP, sensory nerve action potential.

SI conversion factors: To convert total cholesterol, LDL-C, and HDL-C to millimoles per liter, multiply by 0.0259; triglycerides to millimoles per liter, multiply by 1.8; and creatinine to millimoles per liter, multiply by 88.4.

### Lipid Equivalent Lesion Load

**Figure 2.**
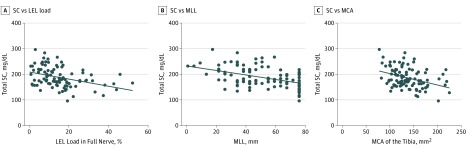
Magnetic Resonance Neurography Sciatic Nerve Findings in Correlation With Serum Cholesterol Levels A, Total serum cholesterol (SC) level vs lipid equivalent lesion (LEL) load. SC level decreased linearly as a function of LEL load (in percentage of nerve tissue) as SC (LEL) = −1.28 mg/dL (%) × LEL + 211.5 mg/dL. B, SC level vs maximum lesion length (MLL). SC level decreased linearly as a function of MLL as SC (MLL) = −4.32 g/dL 1/m × MLL+ 242.50 mg/dL. C, SC level vs mean cross-sectional area (MCA). SC level decreased linearly as a function of the MCA as SC (MCA) = −0.45 × 10^3^ g/dL 1/m^2^ × MCA + 248.40 mg/dL.

We found an increase of T2wFS-hypointense lipid equivalent lesion (LEL) load to be negatively associated with the NCVs of the tibial (*r* = −0.33; *P* = .01) and peroneal nerves (*r* = −0.51; *P* < .001) (eFigure, A and B in the Supplement) and with the CMAPs of the tibial (*r* = − 0.31; *P* = .02) and peroneal nerves (*r* = −0.28; *P* = .03). Further negative correlations were determined between LEL load and total serum cholesterol level (*r* =  −0.41; *P* < .001) (Figure 2A), HDL-C level (*r* = −0.30; *P* = .006), and LDL-C level (*r* = −0.33; *P* = .003). Positive correlations were found between LEL load and the NDS (*r* = 0.31; *P* = .005), NSS (*r* = 0.23; *P* = .03), MCA of the tibial nerve (*r* = 0.44; *P* < .001), and mean maximum length of a lesion (*r* = 0.71; *P* < .001). No significant correlations were found for LEL load and other serologic risk factors, such as HbA_1c_ level, eGFR, creatinine level, or triglyceride levels. Additional partial regression analysis for LEL load and cholesterol levels, controlled for age, disease duration, BMI, eGFR, and creatinine, HbA_1c_, and triglyceride levels, revealed that LEL load was independently correlated with total serum cholesterol (*r* = −0.42; *P* = .001) and LDL-C (*r* = −0.49; *P* < .001) levels.

### Length of Lesions

The mean maximum length of a lesion was positively correlated with the MCA and LEL load. Negative correlations were found for lesion length with NCVs of the tibial (*r* = −0.40; *P* = .002) and peroneal nerves (*r* = −0.47; *P* < .001) (eFigure, C and D in the Supplement) and CMAPs of the tibial (*r* = −0.26; *P* = .049) and peroneal nerves (*r* = −0.26; *P* = .047). Furthermore, we found negative correlations of the lesion length with total serum cholesterol level (*r* = −0.44; *P* < .001) (Figure 2B) and serum LDL-C levels (*r* = −0.38; *P* = .001). Correlations of MRN parameters with clinical, epidemiologic, and serologic factors are given in Table 2 and Table 3. Additional partial regression analysis for the maximum length of a lesion and cholesterol levels, controlled for age, disease duration, BMI, eGFR, and creatinine, HbA_1c_, and triglyceride levels, revealed that the maximum length of a lesion was independently correlated with total serum cholesterol (*r* = −0.42; *P* = .001) and LDL-C (*r* = −0.47, *P* < .001) levels.

**Table 2.** Correlation of Magnetic Resonance Neurography Findings With Clinical and Serologic Data

Variable	Lesion Ratio in Vital Nerve Tissue		Maximum Length of a Lesion		Mean Cross-sectional Area of the Tibial Nerve	
	*r*	*P* Value	*r*	*P* Value	*r*	*P* Value
Lesion ratio in vital nerve tissue	NA	NA	0.71	<.001	0.44	<.001
Maximum length of a lesion	0.71	<.001	NA	NA	0.43	<.001
NSS	0.23	.03	0.02	.86	0.13	.23
NDS	0.31	.005	0.14	.20	0.15	.16
Patient age	0.14	.18	0.10	.36	0.25	.02
BMI	−0.08	.47	−0.02	.89	−0.17	.13
Disease duration	0.21	.06	0.26	.02	0.07	.52
Creatinine level	0.12	.26	0.02	.89	0.19	.07
eGFR	−0.07	.59	0.008	.95	−0.07	.59
Total serum cholesterol level	−0.41	<.001	−0.44	<.001	−0.38	<.001
LDL-C level	−0.33	.003	−0.38	<.001	−0.33	.002
HDL-C level	−0.30	.006	−0.20	.07	−0.20	.08
Triglyceride levels	−0.05	.67	−0.19	.08	−0.08	.67
HbA_1c_	−0.09	.41	−0.02	.86	−0.08	.44
Total serum protein level	0.13	.35	0.10	.50	0.02	.91
Serum albumin level	0.23	.12	0.22	.12	0.02	.90

Abbreviations: BMI, body mass index; eGFR, glomerular filtration rate; HbA_1c_, hemoglobin A_1c_; HDL-C, high-density lipoprotein cholesterol; LDL-C, low-density lipoprotein cholesterol; NA, not applicable; NDS, neuropathy disability score; NSS, neuropathy symptom score.

**Table 3.** Correlation of Magnetic Resonance Neurography Findings With Electrophysiologic and Vascular Data

Variable	Lesion Ratio in Vital Nerve Tissue		Maximum Length of a Lesion		Mean Cross-sectional Area of the Tibial Nerve	
	*r*	*P* Value	*r*	*P* Value	*r*	*P* Value
Sural						
NCV	−0.24	.13	−0.19	.24	−0.40	.01
SNAP	−0.11	.48	−0.27	.08	−0.12	.47
Peroneal						
NCV	−0.51	<.001	−0.47	<.001	−0.46	<.001
CMAP	−0.28	.03	−0.26	.047	−0.48	<.001
DML	0.15	.25	0.19	.15	0.32	.02
Tibial						
NCV	−0.33	.01	−0.40	.002	−0.56	<.001
CMAP	−0.31	.02	−0.26	.049	−0.43	.001
DML	0.09	.49	0.07	.61	0.19	.17
24-h Arterial blood pressure						
Systolic	−0.01	.95	0.02	.91	0.01	.95
Diastolic	−0.04	.85	0.06	.75	−0.08	.67
Mean	−0.04	.82	0.05	.77	−0.09	.64
24-h Pulse	0.21	.25	0.13	.50	−0.08	.65
24-h Arterial night dip						
Systolic	0.12	.52	0.15	.41	0.18	.35
Diastolic	0.12	.54	0.12	.52	0.002	.99
ABI						
Right	−0.15	.29	−0.05	.72	−0.16	.25
Left	−0.22	.13	−0.23	.10	−0.29	.04
IMT						
Right	0.30	.07	0.24	.14	−0.004	.98
Left	0.12	.43	0.19	.20	−0.09	.56

Abbreviations: ABI, ankle-brachial index; CMAP, compound motor action potential; DML, distal motor latency; IMT, intima-media thickness; NCV, nerve conduction velocity; SNAP, sensory nerve action potential.

### MCA of the Proximal Tibial Nerve

We further found the MCA of the proximal tibial nerve to be negatively correlated with NCVs of the tibial (*r* = −0.56; *P* < .001) and peroneal nerves (*r* = −0.46; *P* < .001) (eFigure, E and F in the Supplement) and CMAPs of the tibial (*r* = −0.43; *P* = .001) and peroneal nerves (*r* = −0.48; *P* < .001). An increase of the MCA was negatively correlated with total serum cholesterol (*r* = −0.38; *P* < .001) (Figure 2C) and serum LDL-C (*r* = −0.33; *P* = .002) levels. A positive correlation was found between the MCA and the mean maximum length of a lesion (*r* = 0.44; *P* < .001). Additional partial regression analysis for the MCA and cholesterol levels, controlled for age, disease duration, BMI, eGFR, and creatinine, HbA_1c_, and triglyceride levels, revealed that the MCA was independently correlated with total serum cholesterol (*r* = −0.43; *P* = .001) and LDL-C (*r* = 0.50; *P* < .001) levels.

### Missing Data

Data on the NCV and CMAP of the tibial and/or the peroneal nerve and sural sensory nerve action potentials were missing in a total of 11 participants because patients interrupted the examination or technical problems with the equipment occurred. Another 8 sural nerve sensory nerve action potentials are missing in patients with severe neuropathy and obesity because sensory nerve action potentials could not be recorded properly owing to severe nerve damage or impaired recording caused by patient obesity.

## Discussion

One principle finding of this study was that lipid metabolism may play an essential role in the development of peripheral nerve damage in patients with T2D and DPN. Specifically, we found that low levels of total serum cholesterol and LDL-C were associated with a higher load, diameter, and length of T2wFS-hypointense, lipid-equivalent nerve lesions and with impaired nerve conduction and an increasing severity of a patient’s clinical symptoms. In our cohort, LELs occurred independently from other risk factors, such as elevated HbA_1c_ levels, renal function outcomes, patient’s age, BMI, or disease duration.

To our knowledge, this study was the first to visualize in vivo that low levels of serum cholesterol, specifically LDL-C, were accompanied by peripheral nerve damage in T2D DPN. Our study contradicts the results of previous studies^11,12^ that indicated that lowering serum cholesterol levels potentially slows the progression of DPN by lowering total serum cholesterol and LDL-C levels. Instead, our findings are in line with results of previous studies^15,17,21,29,30^ that found that the intake of statins and a decrease of serum cholesterol level are associated with neuropathic symptoms, microvascular damage, and an accelerated deterioration of peripheral nerve fibers. A potential explanation of the associations found in our cohort might be that lowering serum cholesterol levels impairs peripheral nerve regeneration because cholesterol cannot be produced in axons and therefore has to be supplied to neurite tips and adjacent Schwann cells of regenerating axons by either axonal transport or external supply via HDL-C and LDL-C.^14,18,31,32^ Lowering cholesterol levels with statins has been shown to be associated with a relevant decrease of cholesterol levels available for axonal regeneration on those 2 pathways, resulting in a different composition of lipids in the cholesterol-rich myelin sheath of Schwann cells, which causes nerve swelling attributable to reactive thickening of the myelin sheath comparable to that seen in hereditary disorders in cholesterol metabolism.^14,20^ Thus, an increase of lipid-equivalent nerve lesions and nerve volume, both correlated with a decrease in HDL-C and LDL-C, would represent nerve swelling attributable to an altered composition of lipids in Schwann cells as a consequence of insufficient cholesterol supply to regenerating neurites after neuropathic damage, eventually resulting in decreasing nerve conduction. One possible explanation for the positive associations of lowering lipids with statins with DPN found in previous studies^12,21^ might be that the known antioxidative and anti-inflammatory effects of statins have a positive association with pathophysiologic mechanisms underlying DPN in T2D. In addition, macroangiopathic and microangiopathic changes may contribute to neuropathic damage, thus allowing lipid-lowering therapies to be potentially beneficial for patients with those conditions. One may argue, however, that the association of low serum cholesterol levels with the amount of nerve lesions only occurs as a secondary outcome because patients undergoing lipid-lowering therapies usually have macroangiopathy and microangiopathy and are more likely to have other potential risk factors for DPN, such as hypertension and renal insufficiency. We strove to minimize those potential confounders by excluding all patients with serologic findings of renal insufficiency or macroalbuminuria, and we also excluded all patients with signs of macroangiopathy in the ABI or IMT examination. Furthermore, we found no correlation between LEL load and 24-hour blood pressure recordings. Thus, our results suggest that a low serum cholesterol level is associated with the formation of LELs in T2D DPN. The findings of this study are not completely in line with previous results on the formation of LELs in DPN that have only found a correlation of LEL load with decreasing HDL-C levels but not LDL-C or total serum cholesterol levels in DPN.^10^ However, the aforementioned study comprised a collective of patients with T2D and T1D. The process underlying LEL formation may differ between diabetes types. This study was the first, to our knowledge, to examine the formation of LELs in patients with T2D and DPN exclusively.

The findings are of importance for the understanding of the pathogenetic mechanisms underlying DPN in T2D because the long-term dyslipidemia outcomes seen in humans cannot be properly reproduced in rodents owing to substantial differences in lipid metabolism.^33^ In light of emerging therapies for dyslipidemia in T2D, such as PCSK9 inhibitors that promote a more aggressive lowering of serum cholesterol levels, our results suggest that current clinical trials including patients with very low serum cholesterol levels should pay close attention to signs of neuropathic damage.^16^

### Limitations

This hypothesis-generating study is limited by the fact that only cross-sectional data were acquired, which precludes longitudinal analysis. In addition, our cohort was not equally balanced with male and female participants, which does not allow for sex-specific analyses of our data. Because of the large amount of factors measured, the sample size of 100 participants precludes multivariate analyses with all factors acquired. However, our data are in line with the recently published longitudinal data of the Anglo-Danish-Dutch Study of Intensive Treatment of Diabetes in Primary Care (ADDITION), which found that low levels of HDL-C, total serum cholesterol, and LDL-C were associated with a worsening of DPN.^30^

## Conclusions

This study was the first, to our knowledge, to visualize in vivo that low serum cholesterol levels, especially low LDL-C levels, were associated with peripheral nerve swelling and a higher load of LELs. The associated impaired nerve conduction in patients with T2D and DPN may have been attributable to an impairment of nerve regeneration after neuropathic damage. Regarding novel therapies for treating dyslipidemia in patients with T2D, our results suggest that clinical trials of patients with very low serum cholesterol levels should be vigilant about the onset or deterioration of neuropathic symptoms. Additional longitudinal studies on the role of cholesterol metabolism in DPN appear to be required to determine whether there is a critical threshold of serum cholesterol for an impairment of nerve regeneration.

## Notes

Supplement. **eFigure.** Correlations of MRN Parameters and Electrophysiological Data
