# Supplementary material for: Association of Serum Cholesterol Levels With Peripheral Nerve Damage in Patients With Type 2 Diabetes
Source: JAMA Netw Open. 2019 May 31;2(5):e194798. doi: 10.1001/jamanetworkopen.2019.4798 (PMC6547108; doi:10.1001/jamanetworkopen.2019.4798)
Supplement: Supplement. — eFigure. Correlations of MRN Parameters and Electrophysiological Data [file jamanetwopen-2-e194798-s001.pdf]

Supplementary Online Content

Jende JME, Groener JB, Rother C, et al. Association of serum cholesterol levels with peripheral nerve damage in patients with type 2 diabetes. *JAMA Netw Open*. 2019;2(5):e194798. doi:10.1001/jamanetworkopen.2019.4798

eFigure. Correlations of MRN Parameters and Electrophysiological Data

This supplementary material has been provided by the authors to give readers additional information about their work.

© 2019 Jende JME et al. *JAMA Network Open.*

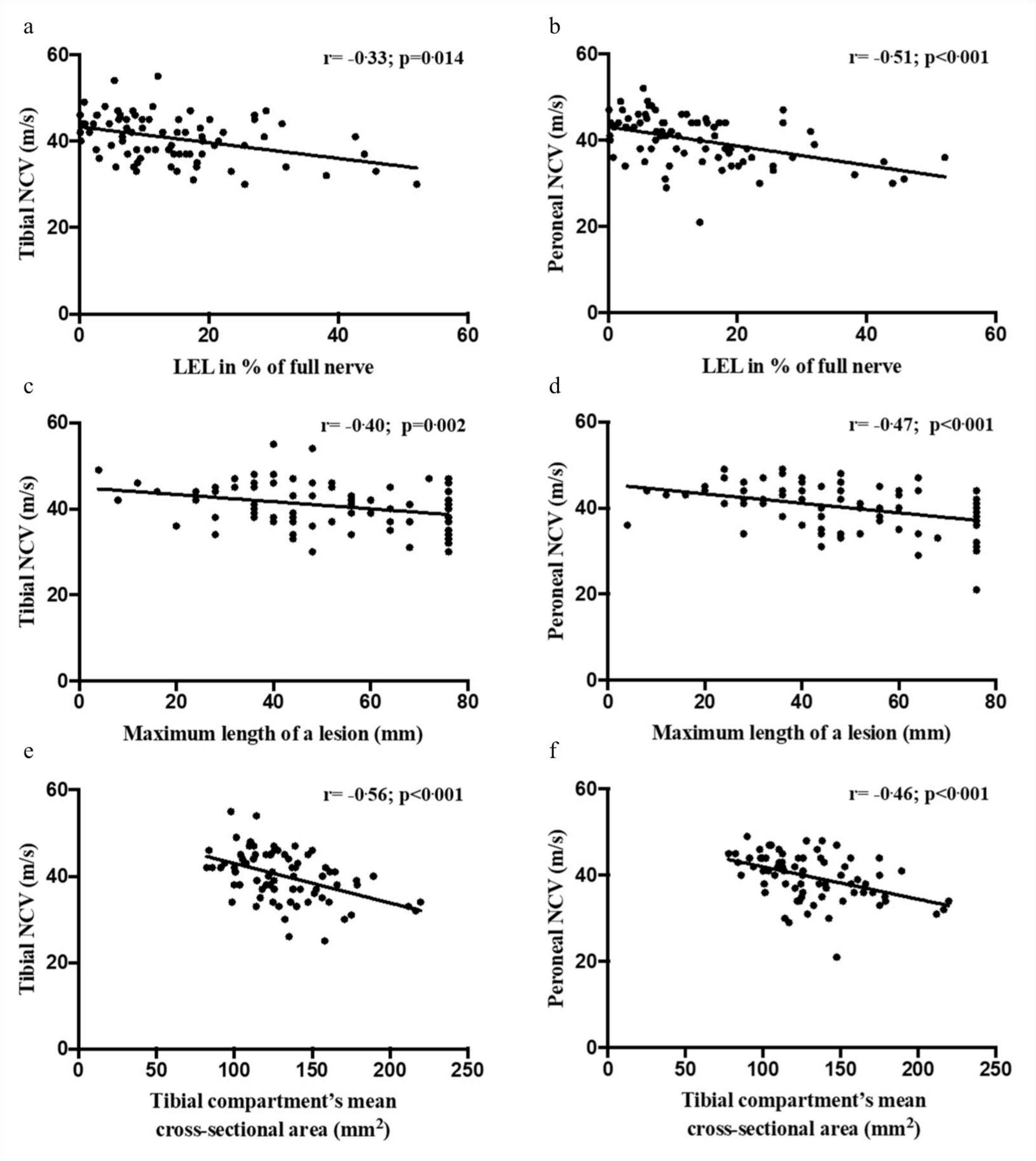

eFigure. Correlations of MRN Parameters and Electrophysiological Data

(a) Peroneal NCV vs. LEL. NCV (in m/s) decreases linearly as a function of LEL (in % of nerve tissue) as NCV(LEL) = -0.22 m/(s %)·LEL + 43.12 m/s. (b) Tibial NCV vs. LEL. NCV (in m/s) decreases linearly as a function of LEL (in % of nerve tissue) as NCV(LEL) = -0.18m/(s %)·LEL + 43.31 m/s. (c) Peroneal NCV vs. maximum length of a lesion (MLL). NCV (in m/s) decreases linearly as a function of MLL (in mm) as NCV(MLL) = -0.11·10^3/s MLL + 45.50 m/s. (d) Tibial NCV vs. MLL. NCV (in m/s) decreases linearly as a function of MLL (in mm) as NCV(MLL) = -0.08·10^3/s MLL + 44.98 m/s. (e) Peroneal NCV vs. tibial compartment mean cross-sectional area (MCA). NCV (in m/s) decreases

© 2019 Jende JME et al. *JAMA Network Open.*

linearly as a function of MCA (in mm^2) as NCV(MCA) = -0.08 10^6/(m s)·MCA + 49.51 m/s. (f) Tibial NCV vs. MCA. NCV (in m/s) decreases linearly as a function of MCA (in mm^2) as NCV(MCA) = -0.092 10^6/(m s)·MCA + 52.17 m/s.
